# How do personality traits modulate real-world gaze behavior? Generated gaze data shows situation-dependent modulations

**DOI:** 10.3389/fpsyg.2023.1144048

**Published:** 2024-01-10

**Authors:** Jumpei Yamashita, Yoshiaki Takimoto, Haruo Oishi, Takatsune Kumada

**Affiliations:** ^1^NTT Access Network Service Systems Laboratories, Nippon Telegraph and Telephone Corporation, Tokyo, Japan; ^2^Graduate School of Informatics, Kyoto University, Kyoto, Japan; ^3^NTT Human Informatics Laboratories, Nippon Telegraph and Telephone Corporation, Kanagawa, Japan

**Keywords:** personality traits, Big Five, gaze behavior, eye and head movements, generative AI, generative adversarial networks, behavior generation, disentanglement

## Abstract

It has both scientific and practical benefits to substantiate the theoretical prediction that personality (Big Five) traits systematically modulate gaze behavior in various real-world (working) situations. Nevertheless, previous methods that required controlled situations and large numbers of participants failed to incorporate real-world personality modulation analysis. One cause of this research gap is the mixed effects of individual attributes (e.g., the accumulated attributes of age, gender, and degree of measurement noise) and personality traits in gaze data. Previous studies may have used larger sample sizes to average out the possible concentration of specific individual attributes in some personality traits, and may have imposed control situations to prevent unexpected interactions between these possibly biased individual attributes and complex, realistic situations. Therefore, we generated and analyzed real-world gaze behavior where the effects of personality traits are separated out from individual attributes. In Experiment 1, we successfully provided a methodology for generating such sensor data on head and eye movements for a small sample of participants who performed realistic nonsocial (data-entry) and social (conversation) work tasks (i.e., the first contribution). In Experiment 2, we evaluated the effectiveness of generated gaze behavior for real-world personality modulation analysis. We successfully showed how openness systematically modulates the autocorrelation coefficients of sensor data, reflecting the period of head and eye movements in data-entry and conversation tasks (i.e., the second contribution). We found different openness modulations in the autocorrelation coefficients from the generated sensor data of the two tasks. These modulations could not be detected using real sensor data because of the contamination of individual attributes. In conclusion, our method is a potentially powerful tool for understanding theoretically expected, systematic situation-specific personality modulation of real-world gaze behavior.

## 1 Introduction

The rationale for this study is the importance of examining how personality traits alter behaviors in real-life situations. Personality traits, including one of the most common sets of traits, the “Big Five,” refer to distinctive and relatively stable characteristics of a person, such as patterns of thoughts, feelings, and behaviors (i.e., behavioral patterns) (Allport and Odbert, 1936; McCrae and Costa, 1987, 2008; Goldberg, 1990). The Big Five describes an individual's personality in five traits: openness, conscientiousness, extraversion, agreeableness, and neuroticism. Recent interactionism emphasizes that these traits systematically modulate behavioral patterns according to different situations (Fleeson, 2004, 2007; Funder, 2006; Baumert et al., 2017; Schmitt and Blum, 2020), which is the theoretical ground of this study. For example, previous studies have shown that extraversion contributes to improved teamwork, while openness promotes broader skill acquisition (Barrick et al., 2001; Wilmot et al., 2019; Laible et al., 2020). The analysis of real-world personality modulation is important because, based on these theories and findings, it may enable the assignment of situations or tasks in a manner that fits individuals' personality traits.

In particular, one promising topic is the analysis of real-world personality modulation of gaze behavior (cf. Kaspar and König, 2012; Kröger et al., 2020). As gaze behavior functions as the fundamental input to cognitive processing (Yarbus, 1967; Just and Carpenter, 1976; Land et al., 1999; Henderson, 2003; Orquin and Loose, 2013), this analysis has revealed how different personalities input visual information for cognitive processing in different ways (Isaacowitz, 2005; Rauthmann et al., 2012; Risko et al., 2012; Lea et al., 2018; Rubo et al., 2023). In line with the interactionist theory, such personality modulations may facilitate the situational behaviors that individuals with certain personalities perform well (e.g., higher openness indicating broader skill acquisition). Therefore, real-world personality modulation analysis of gaze behavior may provide an understanding of the functions of personalities in cognitive processing as well as suggestions for designing situations (e.g., skill training tasks) that suit different personalities.

Previous studies have shown that individuals with higher curiosity, a component of openness, tend to move their gaze with a more liberal basis in scene viewing (Risko et al., 2012). Individuals with higher openness also tend to make longer fixations on abstract animations, reflecting deeper interpretations (Rauthmann et al., 2012). Furthermore, openness has been shown to correlate positively with a stronger preference for face image (Rubo et al., 2023). However, previous methodologies on gaze behaviors have had the shortcomings of controlled experimental situations and large numbers of participants: These methods controlled the situation by presenting fixed or meaningless images at a fixed location (i.e., situation control) to a large number of participants (50*-*250) (Kröger et al., 2020). Since the ideal real-world personality modulation analysis uses realistic situations with only a small number of participants, we do not know whether theoretically expected situation-specific personality modulations are empirically observed in real-world situations. This is the research gap that is addressed by the current study.

**Figure 1:**
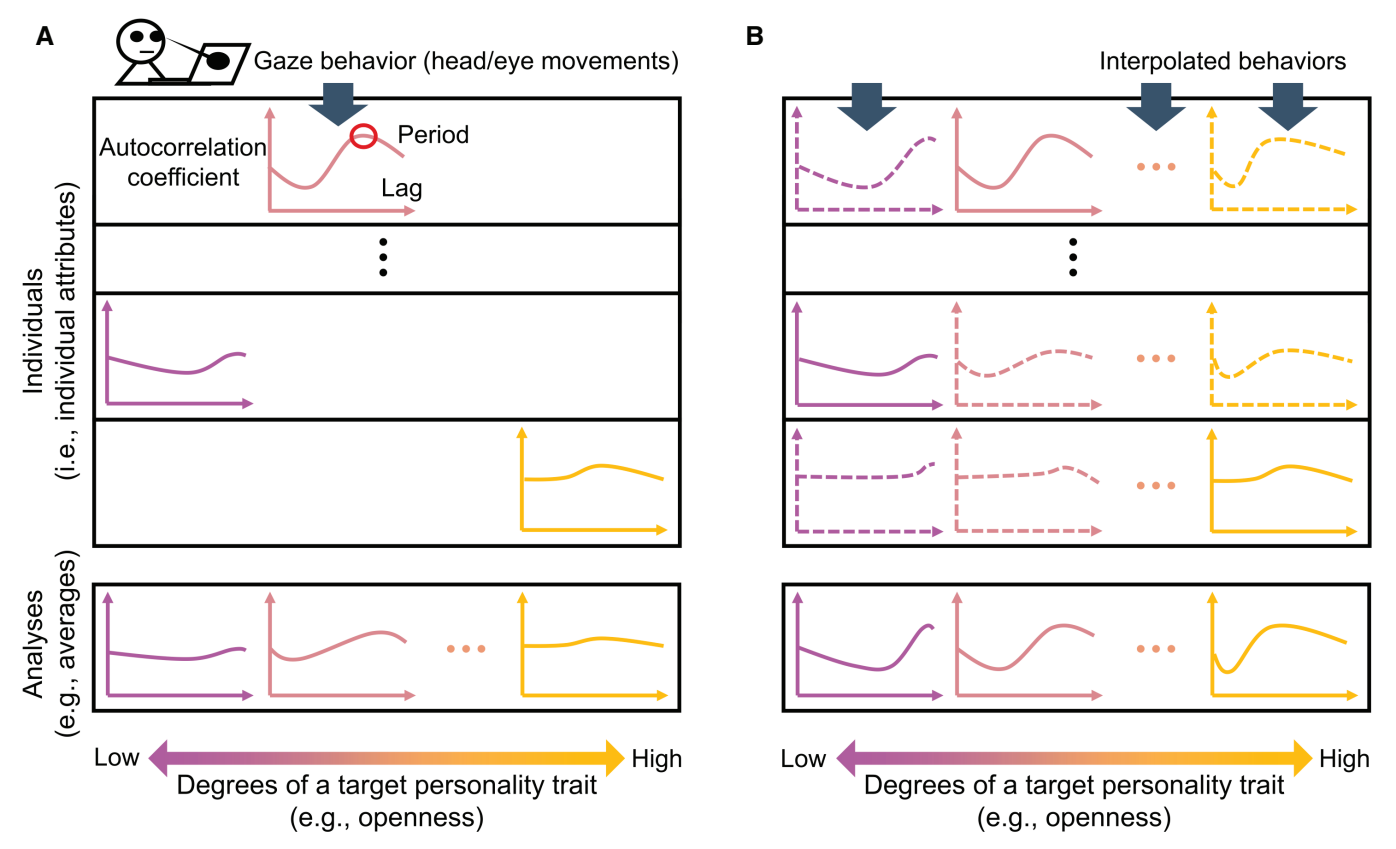
**(A)** Sparse and biased gaze behavior data and **(B)** Interpolated gaze behavior data. The upper two panels of **(A, B)** represent the autocorrelation matrix of the gaze behaviors. The vertical axis represents individuals (individual attributes), and the horizontal axis represents degrees of a target personality trait, as reflected by changes in color. The lower two panels represent the averaging analyses of the gaze behaviors above per degree of that personality trait.

The need for situation control and many participants in previous studies may be attributable to sparse and biased gaze behavior data (Figure 1A). As gaze behavior consists of a repetitive pattern between saccades (i.e., rapid head and eye movements) and fixations (i.e., few/slight movements) (Just and Carpenter, 1976; Bulling et al., 2010; Sağlam et al., 2011), the mean periods of the degree of these movements may reflect the saccade frequency (the mean duration of fixations). If the head and eye movements occur with higher frequency as openness increases (Risko et al., 2012), then higher openness as the target personality trait may lead to a shorter periodicity, i.e., the time interval (lag) at which the autocorrelation coefficient peaks, across individuals. In contrast, individual attributes, such as demographic attributes and the state of the measurement device for each participant, may change the entire autocorrelation pattern of each individual discontinuously (cf. Vinciarelli and Mohammadi, 2014; Junior et al., 2019; Phan and Rauthmann, 2021). In that case, the autocorrelation matrix of target personality trait degrees and individuals (i.e., individual attributes uniquely accumulated in individuals) should be sparse and could be biased, making target personality-related differences undetectable. Previous studies may have used larger sample sizes to average out the sparsity-related bias, such as the concentration of specific individual attributes in some personality traits, and may have controlled the experimental scenarios to prevent unexpected interactions between these possibly biased individual attributes and complex, realistic situations.

To fill the research gap, an effective procedure would generate gaze behavior data interpolated from the minimum to the maximum degree of the target personality trait for all individuals (Figure 1B). With such an interpolated dataset, we could, for example, compare differences in the mean periods of the head and eye movements attributable to degrees of the target personality trait (e.g., openness), while excluding the possibility of biased differences in the entire autocorrelation patterns due to individuals. Accordingly, we could extract target personality-related differences even with a few participants in realistic situations. This may be understood by comparing it to building a machine learning model to classify wolves, which often appear in snowy landscapes, and huskies, which often appear in sunny landscapes, in photographs (Ribeiro et al., 2016). To train a model that extracts the feature differences between the animals, instead of the landscapes, we may manually control the numbers of snowy and sunny landscapes for wolves and huskies, or synthesize the wolves and huskies in all the snowy and sunny landscapes, before training (Zhang et al., 2018). The “synthesis” or generation, is helpful in our case because the “landscapes,” or individual attributes, are not easily manipulated.

**Figure 2:**
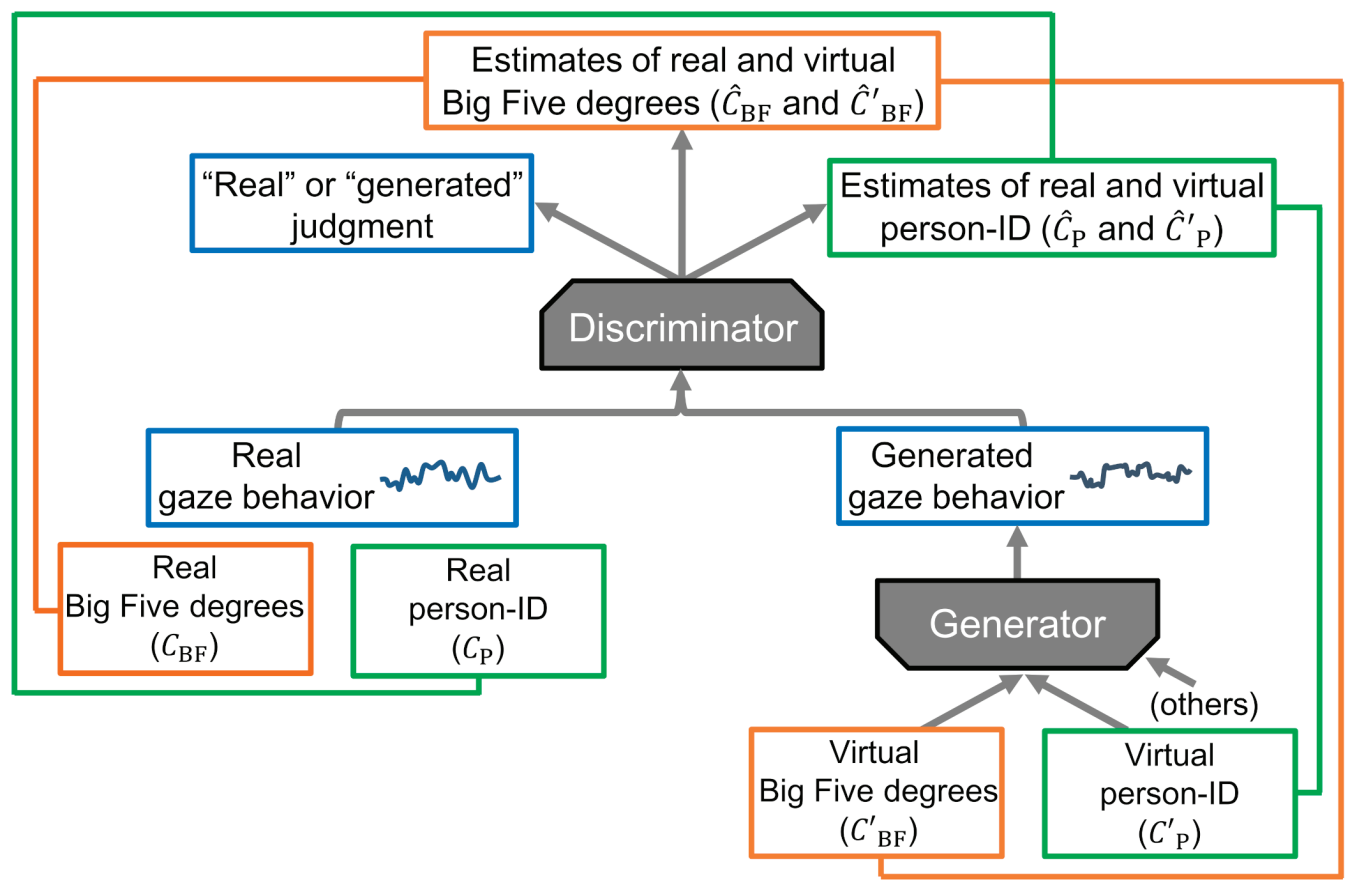
Overview of our ss-InfoGAN. Squares represent the data (behavior data or labels), arrows represent the data flow, and lines connecting the two data represent pieces of estimation training that bring the two label values closer together.

The first contribution of the current study is to provide a methodology for generating interpolated gaze behavior. Experiment 1 constructed and evaluated semi-supervised Information Maximizing Generative Adversarial Networks (ss-InfoGAN) (Goodfellow et al., 2014; Chen et al., 2016; Spurr et al., 2017; Lin et al., 2020) (Figure 2). Our ss-InfoGAN consists of a generator, which generates gaze behavior from latent codes of the Big Five (i.e., labels of personality traits) and person-IDs (i.e., labels of individuals), and a discriminator, which discriminates between generated and real gaze behavior. Whereas the discriminator learns to correctly identify whether input gaze behavior is “real” or “generated,” the generator learns to output gaze behavior that deceives the discriminator to make judgments of “real” (Goodfellow et al., 2014). This adversarial training enables the generator to generate gaze behavior similar to real data. Further, the discriminator outputs estimates of the real Big Five degree labels (*ĉ*_BF_) and estimates of the real person-ID labels (*ĉ*_P_) from real gaze behavior; these estimates are trained to be close to the real Big Five degree labels (*c*_BF_) and the real person-ID labels (*c*_P_). We also trained the generator so that the continuous and categorical latent codes ($c_{\text{BF}}^{\prime }$ and $c_{\text{P}}^{\prime }$), which are the inputs to generate gaze behavior, could be correctly estimated (${ĉ}_{\text{BF}}^{\prime }$ and ${ĉ}_{\text{P}}^{\prime }$) in the output path of the estimates of the real Big Five degrees (*ĉ*_BF_) and the real person-ID labels (*ĉ*_P_) in the discriminator. As a result, we expected to generate gaze behavior while virtually changing the Big Five degrees (i.e., the virtual version of the Big Five degrees; $c_{\text{BF}}^{\prime }$) and the individuals (i.e., the virtual versions of the person-ID; $c_{\text{P}}^{\prime }$), resulting in the interpolated gaze behavior (Chen et al., 2016; Spurr et al., 2017).

The second contribution of the current study is to evaluate the effectiveness of interpolated gaze behavior for real-world personality modulation analysis of a small number of participants in realistic situations. In line with the interactionist theory, Experiment 2 examined whether the expected situation-dependent personality modulations would be more clearly observed in the interpolated gaze behavior than in the real gaze behavior. We analyzed the autocorrelation coefficient of head and eye movements as an example of the feature that could be tested for interpretation consistent with previous studies, cf. openness-related differences in the saccade frequency (the fixation duration) (Rauthmann et al., 2012; Risko et al., 2012).

The remainder of this paper is organized as follows. Section 2 presents Experiment 1, which provides the method for generating interpolated gaze behavior and the results of its evaluation (i.e., the first contribution of the present study). Section 3 describes Experiment 2, which evaluates the effectiveness of interpolated gaze behavior in real-world personality modulation analysis (the second contribution). Section 4 presents the discussion and conclusions. Note that this paper is a revised version of Chapter 4 of the first author's doctoral dissertation (Yamashita, 2023).

## 2 Experiment 1

To address the research gap, Experiment 1 first provides a methodology for generating interpolated gaze behavior (the first contribution). For this purpose, we first collected gaze behavior in a realistic office-work setting, which was obtained from relatively small samples of 10*-*20 people. In contrast to previous studies using controlled experiments that required 50*-*250 participants, our participants performed realistic nonsocial (i.e., data entry) and social (i.e., conversation) tasks (Dotti et al., 2018) in which they were expected to exhibit different gaze behavior depending on their personality traits (Isaacowitz, 2005; Rauthmann et al., 2012; Risko et al., 2012; Lea et al., 2018; Rubo et al., 2023). The number of participants was small, falling within the range of a typical eye movement study (Winkler and Subramanian, 2013).

We measured the Big Five traits of participants using a Japanese sentence-based self-questionnaire, the Trait Descriptors Personality Inventory (TDPI) (Iwai et al., 2019). We also measured time series data of head and eye movements from a commercial wearable device called JINS MEME, which has head-mounted motion sensors (i.e., accelerometers and gyrometers) and eye movement sensors (i.e., electrooculography) (Ishimaru et al., 2014).

With this dataset, we then constructed and evaluated ss-InfoGAN (Chen et al., 2016; Spurr et al., 2017) to generate interpolated gaze behavior (sensor data). Given the definitions of interpolated sensor data, we conducted three tests—*real data test, generated data test*, and *real and generated data test*—to evaluate whether generated feature changes that depend on the virtual target personality trait ($c_{\text{BF}}^{\prime }$) within each participant could mimic real feature changes acquired from real participants with different degrees of that trait (*c*_BF_) (cf. Figures 1, 2) (Salimans et al., 2016; Spurr et al., 2017). Since analysis could not proceed without Big Five modulation of the collected sensor data, the real data test first examined whether there was a modulation of features by the target personality trait (*c*_BF_) in the real sensor data. We did not have a strong hypothesis about modulation by the real Big Five because few previous studies used realistic tasks. Nevertheless, openness, in particular, may modulate sensor data because openness (or curiosity) has been reported to significantly affect gaze behavior in multiple laboratory-controlled studies (Rauthmann et al., 2012; Risko et al., 2012; Rubo et al., 2023). The generated data test then examined whether there was modulation of features by the virtual target personality trait ($c_{\text{BF}}^{\prime }$) in the generated sensor data. The sensor data generated while changing the virtual target personality trait should have specific and detectable feature changes (Spurr et al., 2017). Finally, the real and generated data test examined whether the above two modulations shared similarities. The features of real sensor data modulated by the real target personality trait (*c*_BF_) should be similar to those of the generated sensor data modulated by the virtual target personality trait ($c_{\text{BF}}^{\prime }$).

In these three tests, we calculated Pearson's correlation coefficient r, which is most commonly used as an accuracy indicator in detecting relative, not absolute, personality modulation of behaviors (Chang and Lin, 2011; Phan and Rauthmann, 2021). A ceiling of around *r* ≈ 0.30*-*0.50 for correlations between estimated and measured personality is typical in this field (Phan and Rauthmann, 2021). Therefore, we expected to obtain positive correlations within this range in these tests. We performed statistical hypothesis testing on correlations in the real data but not on correlations in the generated data. Since the sample size of the generated data is arbitrarily large, it is not appropriate to apply a statistical test, but it is appropriate to report descriptive statistics to show the effect size (Lantz, 2013).

### 2.1 Materials and methods

#### 2.1.1 Data collection

##### 2.1.1.1 Participants

Participants in the experiment were university students with normal or corrected-to-normal vision (*N* = 20, 10 women; age range: 19*-*25 years [mean 21.9 years]). We obtained valid sensor data from 14 participants, because battery failure in the sensor device prevented proper data transfer for 6 participants (*N* = 14, 6 women, mean 21.8 years).^1^ All received payment for their participation. The studies involving human participants were reviewed and approved by the Ethics Committee, Graduate School of Informatics, Kyoto University (KUIS-EAR-2019-004). The patients/participants provided their written informed consent to participate in this study. Note that we used data from our previous paper (Yamashita et al., 2022). See also the author's note.

##### 2.1.1.2 Apparatus and stimuli

We collected Big Five and sensor data on the head and eye movements of the participants. We measured the Big Five by using TDPI (Iwai et al., 2019). The participants rated their degree of fit to 20 questions on a seven-point scale, with four questions for each trait (Iwai et al., 2019). We used the JINS MEME wearable device (Ishimaru et al., 2014), which is similar in size to ordinary glasses, to obtain 50 Hz sensor data on head movements (accelerometers: ACC X, Y, and Z; gyrometers: GYRO X, Y, and Z) and eye movements (absolute potential of left electrode: EOG L; vertical potential difference: EOG V; horizontal potential difference: EOG H). Since our goal was to analyze participants in realistic situations, we did not impose strong controls on the placement of the stimuli or the posture of the participants. In the data-entry task, participants were seated approximately 60 cm from a 59 × 33 cm monitor.

##### 2.1.1.3 Procedure and design

Each participant performed four tasks, *data entry, conversation, baseline*, and *proofreading*, and performance on the former three was analyzed for this study. Each task took about ten minutes. The total procedure lasted approximately 60 min, including task preparation. The fourth task required participants to read documents using controlled stimuli, and these data were not used in the current study. All tasks were performed in a random order, except that the conversation task was fixed at the end because the topic of the conversation task was how participants felt about the other tasks.

In the nonsocial (data entry) task, participants were instructed to post data from paper accounting documents to files in a spreadsheet program on a PC. Specifically, they were instructed to enter as many text elements as possible, such as a company's name, income, and spending from different paper forms into appropriate positions in the table files. In the social (conversation) task, the participants were instructed to converse with an experimenter about their impressions of the experiment. No participants knew the experimenter personally, and the participants' goal was essentially to pass the time with safe small talk until the set time had elapsed. This conversation task was intended to be similar to an interaction with others in the workplace in which the interacting people are not so close to each other.

In the baseline task, the participants were instructed to freely pass the time alone in the laboratory during a break. We assumed that there would be no Big Five modulation of these sensor data because of the lack of a common situation (see Section 1). Using this task, we confirmed that the results of the social and nonsocial tasks were not derived from a situation in which the Big Five could be estimated by any data input.

#### 2.1.2 Data generation

**Figure 3:**
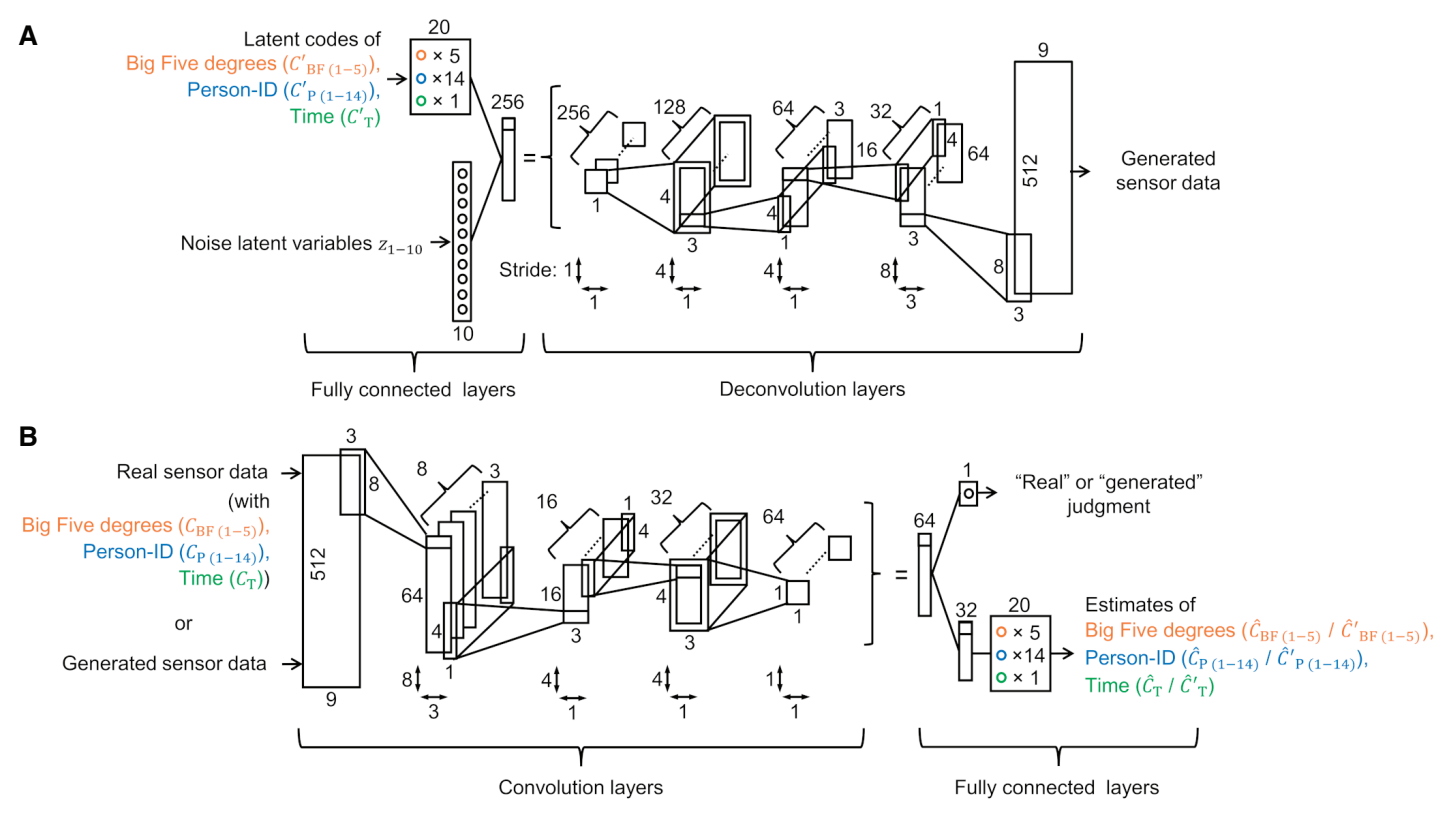
**(A)** Generator architecture and **(B)** discriminator architecture.

We constructed an ss-InfoGAN to generate interpolated data by disentangling the relationships among the sensor data, the personality traits (Big-Five labels, *c*_BF(1-5)_ and $c_{\text{BF}\left(1\text{-}5\right)}^{\prime }$), the individual attributes (person-ID labels, *c*_P(1-14)_ and $c_{\text{P}\left(1\text{-}14\right)}^{\prime }$), and the elapsed time on a task (time labels, *c*_T_ and $c_{\text{T}}^{\prime }$) (Figures 2, 3). Through this method, we could estimate the degrees of the real Big Five (*c*_BF(1-5)_) from the real sensor data and also generate sensor data while changing the degrees of the virtual Big Five ($c_{\text{BF}\left(1\text{-}5\right)}^{\prime }$). The former function was used for validation of the model, while the latter function was used for our main purpose. Note that the time labels (*c*_T_ and $c_{\text{T}}^{\prime }$) were added as a latent code only to provide sufficient variation in the generated data. The codes on which our implementation was based can be found on GitHub (Chen et al., 2016; Lin et al., 2020).^2^ We modified these implementations, which were originally used for image datasets, as described below.

##### 2.1.2.1 Input (real data) preprocessing

The smoothed JINS MEME 50 Hz real sensor data from head and eye movements were divided using a window of 512 points (10.24 seconds) that moved in increments of 128 points (2.56 seconds). That is, we obtained 10.24-second pieces of 50 Hz sensor data in which eye movement events (e.g., saccades and fixations) could be observed (Ishimaru et al., 2014, 2016, 2017). Input data in arrays of 9 × 512 (for the sensor data modality and time, respectively) were then standardized for each modality, each participant, and each task to have a mean of 0 and a standard deviation of 1.

We used the following three types of labels along with each piece of sensor data. First, the real Big-Five labels (*c*_BF(1-5)_) consisted of standardized Big Five values, with a mean of 0 and a standard deviation of 1 within each trait. Second, the real person-ID labels (*c*_P(1-14)_) consisted of one-hot vectors of the number of participants (14 in this study). Finally, the real time labels (*c*_T_) indicated the elapsed time on a task; here, the start and end points of a task were set to -1 and 1, respectively, and the values were meant to increase evenly.

##### 2.1.2.2 Model architecture

The generator consisted of one fully connected layer and four deconvolution layers, as shown in Figure 3A. The input, which consisted of arrays of 20 latent codes (5 for Big-Five: $c_{\text{BF}\left(1\text{-}5\right)}^{\prime }$; 14 for person-ID: $c_{\text{P}\left(1\text{-}14\right)}^{\prime }$; and 1 for time: $c_{\text{T}}^{\prime }$) and arrays of 10 noise latent variables (*z*_1*-*10_), was transformed into multi-dimensional (9 × 512) generated sensor data. The generator used the ReLU activation function (Radford et al., 2016) except for the output layer that used the linear function. Batch normalization was used except for the output layer (Radford et al., 2016).

Figure 3B shows the discriminator, which consisted of four convolution layers followed by one or two fully connected layers. The input data of the multi-dimensional (9 × 512) real or generated sensor data were transformed into a one-dimensional output that discriminated between real or generated data and 20 outputs that were estimates by the three types of real labels (*ĉ*_BF(1-5)_, *ĉ*_P(1-14)_, and *ĉ*_T_) of the real sensor data input or estimates by the latent codes (${ĉ}_{\text{BF}\left(1\text{-}5\right)}^{\prime }$, ${ĉ}_{\text{P}\left(1\text{-}14\right)}^{\prime }$, and ${ĉ}_{\text{T}}^{\prime }$) of the generated sensor data input. In the convolution layers, reflection padding was used (Zhu et al., 2017). LeakyReLU activation function (Maas et al., 2013; Xu et al., 2015) was used except for the output layer, with multiplication by 0.2 when the input value range was less than zero (Radford et al., 2016). The activation functions were sigmoid for discriminating between generated and real data, linear for estimating the continuous latent codes (*ĉ*_BF(1-5)_, ${ĉ}_{\text{BF}\left(1\text{-}5\right)}^{\prime }$, *ĉ*_T_, and ${ĉ}_{\text{T}}^{\prime }$), and softmax for estimating the multi-categorical latent codes (*ĉ*_P(1-14)_ and ${ĉ}_{\text{P}\left(1\text{-}14\right)}^{\prime }$) (Chen et al., 2016; Lin et al., 2020). Spectral normalization was used for all layers except the output layer (Miyato et al., 2018).

##### 2.1.2.3 Model training

Using data in a random order, we performed 200 epochs of training with a mini-batch size of 256. We used Adam (Kingma and Ba, 2014; Radford et al., 2016) with the “translation” model construction technique (Zhao et al., 2020) to minimize loss. Adam was used with α = 0.005, β_1_ = 0.5, β_2_ = 0.9 for the discriminator and α = 0.001, β_1_ = 0.5, β_2_ = 0.9 for the generator (Heusel et al., 2017). Binary cross-entropy loss was used for discriminating between generated and real data, negative log-likelihood loss was used for estimating the continuous latent codes (*ĉ*_BF(1-5)_, ${ĉ}_{\text{BF}\left(1\text{-}5\right)}^{\prime }$, *ĉ*_T_, and ${ĉ}_{\text{T}}^{\prime }$), and cross-entropy loss was used for estimating the multi-categorical latent codes (*ĉ*_P(1-14)_ and ${ĉ}_{\text{P}\left(1\text{-}14\right)}^{\prime }$) (Chen et al., 2016; Lin et al., 2020). To improve the generated data quality, we incorporated a translation technique in which, for 50% of the real and generated data, we divided the time series of sensor data at random points and flipped one of them in the time direction (Zhao et al., 2020). We minimized the term of the mean squared difference in accuracy between the original and translated data (Zhao et al., 2020). The detailed training procedure is as follows.

In each epoch, we optimized first the discriminator and then the generator. To optimize the discriminator, we first generated the sensor data by using the generator with latent codes of $c_{\text{BF}\left(1\text{-}5\right)}^{\prime }$, $c_{\text{P}\left(1\text{-}14\right)}^{\prime }$, and $c_{\text{T}}^{\prime }$ and noise latent variables *z*_1*-*10_ as the input. Latent codes $c_{\text{BF}\left(1\text{-}5\right)}^{\prime }$ (Big-Five) were sampled from a normal distribution with a mean of 0 and a standard deviation of 1. The one-hot vectors for latent codes $c_{\text{P}\left(1\text{-}14\right)}^{\prime }$ (person-ID) were sampled randomly with the probability of 1/14 for each unit to take the value 1. Finally, the latent code $c_{\text{T}}^{\prime }$ (time) and the noise latent variables *z*_1*-*10_ were sampled from a uniform distribution with a range of [−1, 1]. The generated sensor data were input to the discriminator, and in the path for discriminating between generated and real data, back-propagation was done so that the output approached 0 (i.e., “generated”). Next, the real data was input to the discriminator, and back-propagation was done so that the output approached 1 (i.e., “real”). In the path for estimating the latent codes, back-propagation was done so that the output (*ĉ*_BF(1-5)_, *ĉ*_P(1-14)_, and *ĉ*_T_) approached the degrees of the real Big-Five labels (*c*_BF(1-5)_), the person-ID labels (*c*_P(1-14)_), and the time labels (*c*_T_).

In optimizing the generator, sensor data were generated with the same generation process used in optimizing the discriminator. In the path for discriminating between generated and real data, back-propagation was done so that the output approached 1 (i.e., “real”). In the path for estimating the latent codes, the error was back-propagated so that the output (${ĉ}_{\text{BF}\left(1\text{-}5\right)}^{\prime }$, ${ĉ}_{\text{P}\left(1\text{-}14\right)}^{\prime }$, and ${ĉ}_{\text{T}}^{\prime }$) approached latent codes ($c_{\text{BF}\left(1\text{-}5\right)}^{\prime }$, $c_{\text{P}\left(1\text{-}14\right)}^{\prime }$, and $c_{\text{T}}^{\prime }$), which were the source of the generated sensor data in the generator. In addition to the generator, training was performed on the two fully connected layers in the discriminator that were relevant for output of the latent codes (${ĉ}_{\text{BF}\left(1\text{-}5\right)}^{\prime }$, ${ĉ}_{\text{P}\left(1\text{-}14\right)}^{\prime }$, and ${ĉ}_{\text{T}}^{\prime }$).

**Figure 4:**
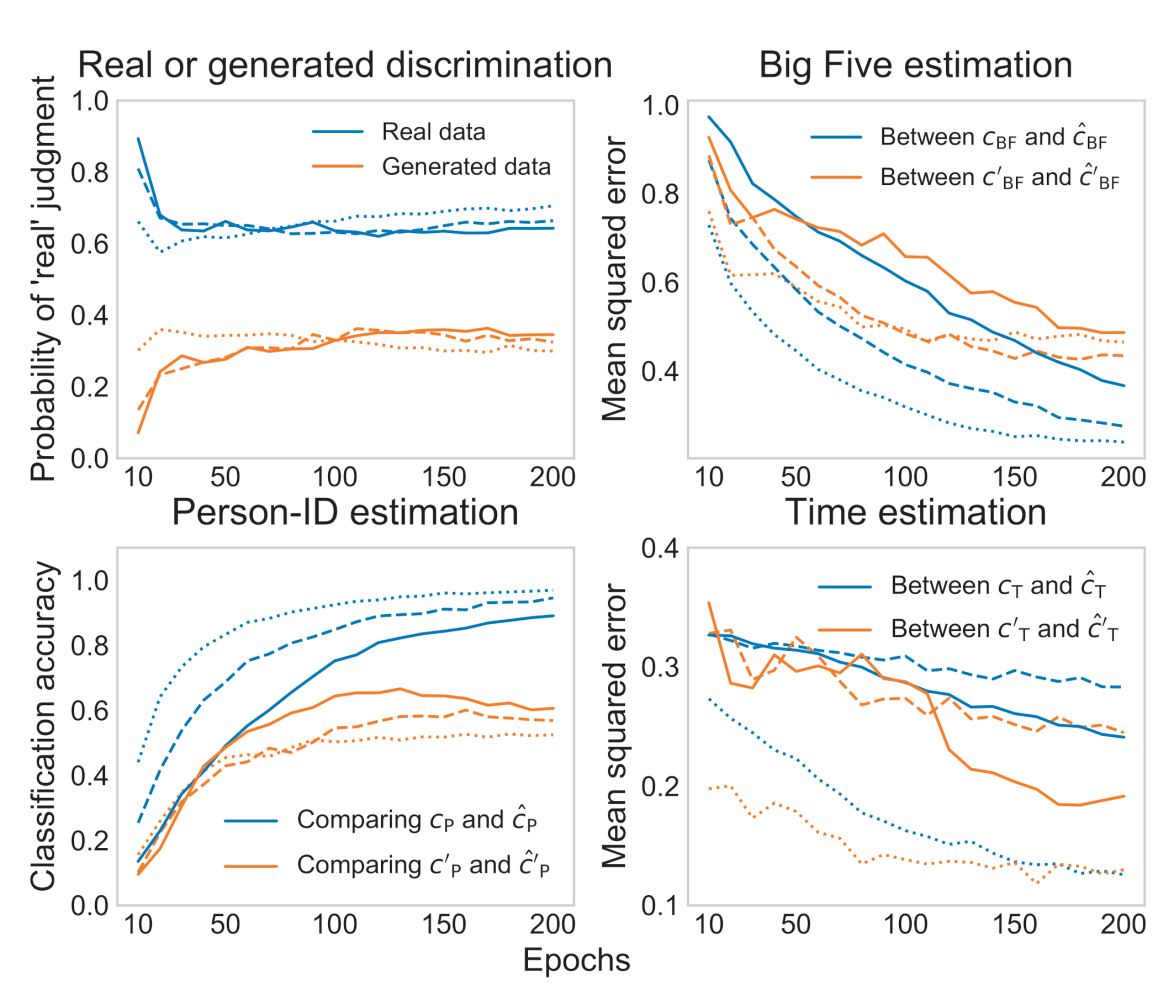
Training errors (solid line: data entry; dashed line: conversation; dotted line: baseline).

Figure 4 shows the errors in the training processes. We found that, in the later epochs, the probabilities of “real” judgment for the real data (60*-*70%) and for the generated data (30*-*40%) were stable, which suggests that adversarial training was established. We also generally found significant decreases in training errors for latent codes ${ĉ}_{\text{BF}\left(1\text{-}5\right)}^{\prime }$ and ${ĉ}_{\text{P}\left(1\text{-}14\right)}^{\prime }$ and the Big-Five and person-ID labels (*ĉ*_BF(1-5)_ and *ĉ*_P(1-14)_); the only exception was the latent code ${ĉ}_{\text{T}}^{\prime }$ and the time label (*ĉ*_T_), whose role was only to increase the variation in the generated data. Accordingly, the model construction progressed appropriately.

### 2.2 Results

We designed and conducted three tests: real data test, generated data test, and real and generated data test (cf. the tested data and labels in Figure 3) (Salimans et al., 2016; Spurr et al., 2017). First, we expected modulation of the real sensor data features by the real Big Five labels (*c*_BF(1-5)_), especially by openness (*c*_BF(1)_). To examine this, we trained the whole ss-InfoGAN on the real sensor data and a limited number of real labels that excluded the real Big-Five labels of one participant, we input the real sensor data of the excluded participant to the trained discriminator, and we compared the actual values of *c*_BF(1-5)_ and the output estimated values of *ĉ*_BF(1-5)_ for that excluded participant. This process was repeated 14 times until all participants had been excluded (i.e., leave-one-out cross-validation: LOOCV) (Yarkoni and Westfall, 2017). We then examined whether there was a significant positive, between-individual correlation coefficient r between the actual (*c*_BF(1-5)_) and estimated (*ĉ*_BF(1-5)_) degrees of the real Big Five labels. If there was a modulation of real sensor data features by the target personality trait (*c*_BF(1-5)_), the ss-InfoGAN should extract those relationships, and therefore we should see a significant positive correlation between the actual (*c*_BF(1-5)_) and estimated (*ĉ*_BF(1-5)_) degrees of the real Big Five labels in the real data test.

**Figure 5:**
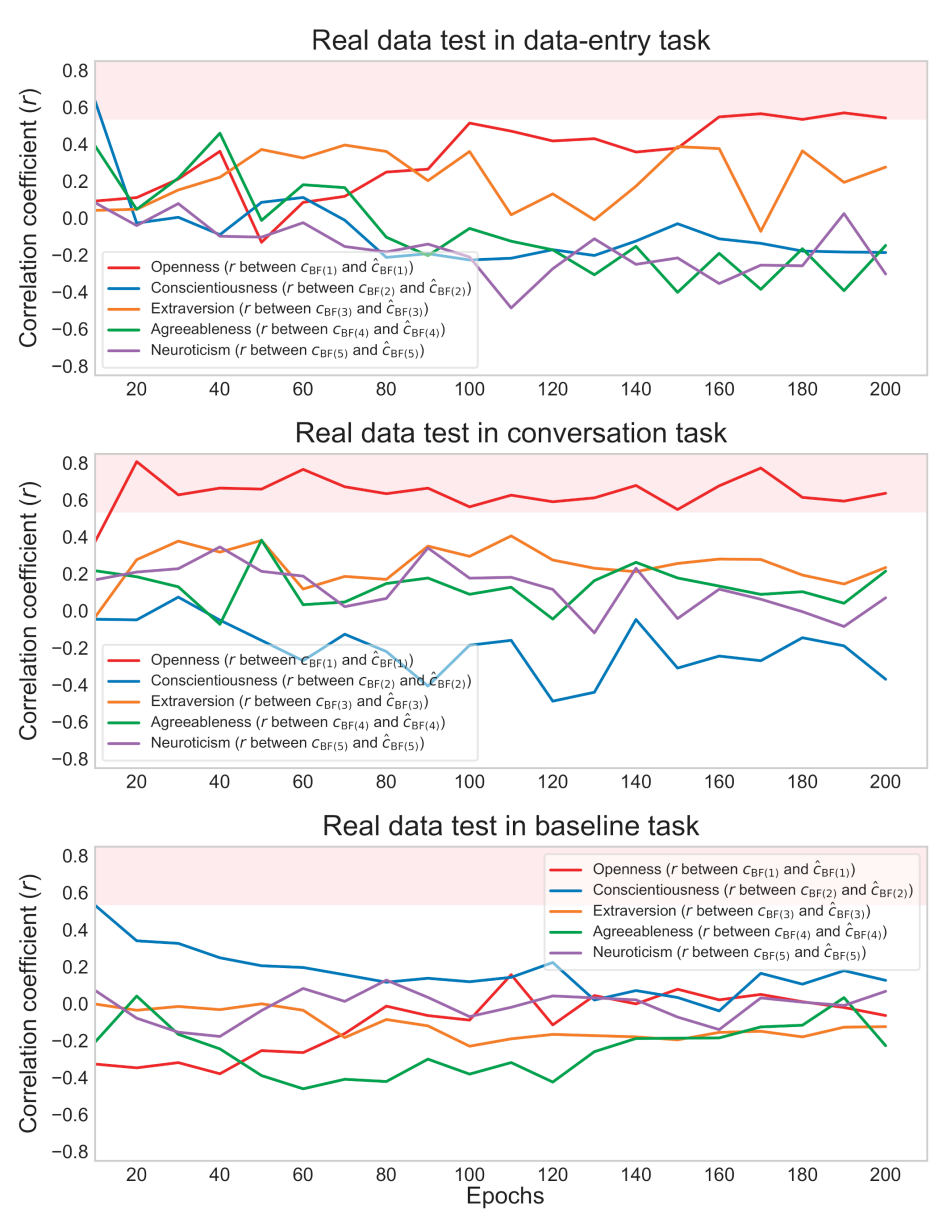
Correlation coefficients in data-entry, conversation, and baseline tasks in the real data test. The red background indicates the region of significant positive correlation.

**Figure 6:**
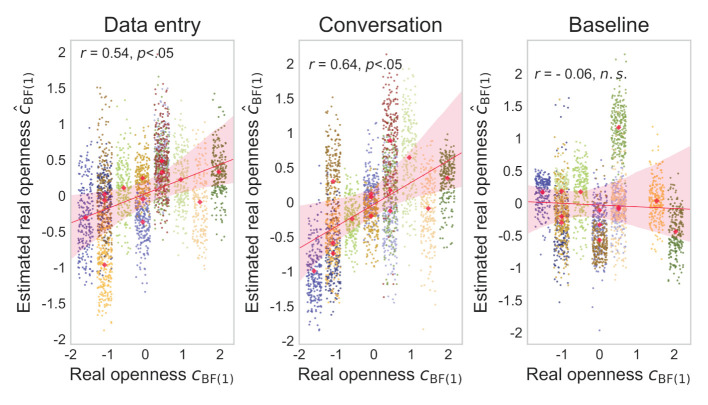
Scatterplots for data-entry, conversation, and baseline tasks in the real data test. Small points represent openness values for each piece of sensor data, and different colors represent points of different individuals. The variability of the measured values within the same individual was due to random noise. Red points represent averaged openness values for each individual.

For the data-entry and conversation tasks, the actual (*c*_BF(1)_) and estimated (*ĉ*_BF(1)_) degrees of real openness showed a significant between-individual positive correlation of about *r* = 0.6 in the later epochs of the real data test (Figure 5). On the other hand, for the baseline task, the actual (*c*_BF(1-5)_) and estimated (*ĉ*_BF(1-5)_) degrees for all the Big Five labels showed no significant correlations (all *ps* > 0.05). Scatterplots of the actual (*c*_BF(1)_) and estimated (*ĉ*_BF(1)_) degrees of openness in the final epoch are shown in Figure 6. In the subsequent analysis, only significant relationships with openness are reported.

Second, we expected modulation of the generated sensor data features by virtual openness ($c_{\text{BF}\left(1\right)}^{\prime }$). To examine this, we conducted the generated data test. The training procedure was carried out using the data of all participants. In light of our definition of interpolated data (Figure 1B), we evaluated the sensor data generated while keeping person-ID ($c_{\text{P}\left(1\text{-}14\right)}^{\prime }$) constant and changing the target personality trait of openness ($c_{\text{BF}\left(1\right)}^{\prime }$). Sensor data was thus generated by simulating 100 cases in which each participant had randomly different degrees of virtual openness ($c_{\text{BF}\left(1\right)}^{\prime }$) (100 × 14 cases). We calculated within-individual correlation coefficients between the actual ($c_{\text{BF}\left(1\right)}^{\prime }$) and estimated (${ĉ}_{\text{BF}\left(1\right)}^{\prime }$) degree of virtual openness for each participant. If there was modulation of the generated sensor data features by virtual openness ($c_{\text{BF}\left(1\right)}^{\prime }$), the ss-InfoGAN should extract those relationships, and therefore we should see a strong positive correlation between the actual ($c_{\text{BF}\left(1\right)}^{\prime }$) and estimated (${ĉ}_{\text{BF}\left(1\right)}^{\prime }$) degrees of virtual openness in the generated data test.

**Figure 7:**
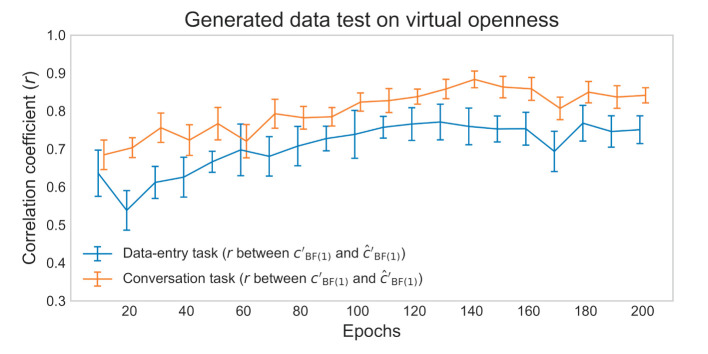
Mean correlation coefficients for data-entry and conversation tasks in the generated data test. Error bars represent standard deviations (SD).

**Figure 8:**
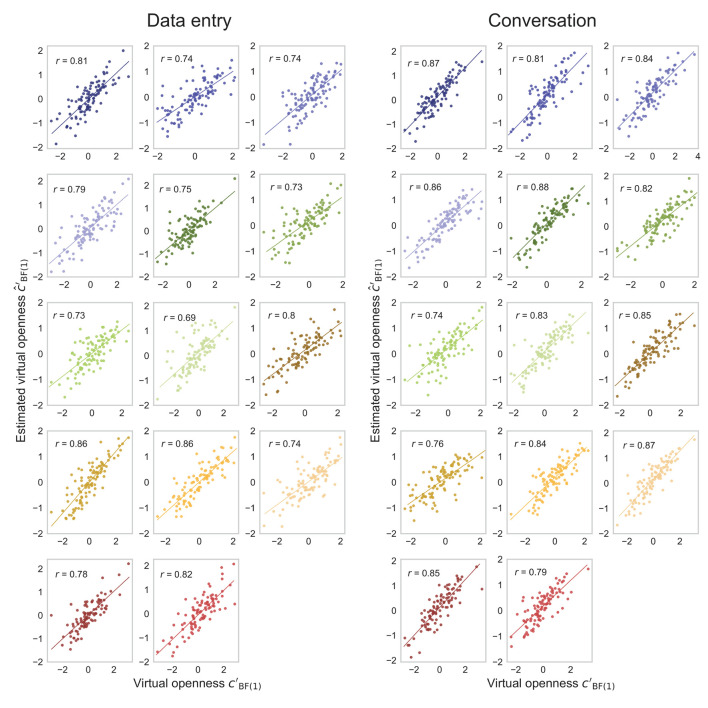
Fourteen scatterplots for data-entry and conversation tasks in the generated data test. Points represent openness values for each piece of sensor data, and different colors represent points of different individuals.

The actual ($c_{\text{BF}\left(1\right)}^{\prime }$) and estimated (${ĉ}_{\text{BF}\left(1\right)}^{\prime }$) degrees of virtual openness showed a strong within-individual positive correlation of about *r* = 0.7*-*0.8 in the later epochs of the generated data test (Figure 7). These results suggest that the generated sensor data changed depending on virtual openness ($c_{\text{BF}\left(1\right)}^{\prime }$) even within each individual. Scatterplots of the actual ($c_{\text{BF}\left(1\right)}^{\prime }$) and estimated (${ĉ}_{\text{BF}\left(1\right)}^{\prime }$) degrees of virtual openness in the final epoch are shown in Figure 8 (data entry: mean *r* = 0.77 [*SD* = 0.05]; conversation: mean *r* = 0.83 [*SD* = 0.04]). No statistical tests were performed because it is not appropriate to conduct statistical tests on arbitrarily large sample sizes (see Section 2, Paragraph 4).

Finally, changes in the generated sensor data that depend on the degree of virtual openness ($c_{\text{BF}\left(1\right)}^{\prime }$) should mimic the real sensor data differences obtained from participants with different degrees of real openness (*c*_BF(1)_). To examine this, we conducted the real and generated data test. We prepared an additional, untrained model with the same architecture as the discriminator. We trained this model only to estimate the degrees of real openness (*c*_BF(1)_) from the real sensor data on an individual basis using the same optimization setting as the discriminator (except for the estimation output). That is, we obtained a model that outputs the estimated real openness (*ĉ*_BF(1)_) using the actual real openness of the real sensor data (*c*_BF(1)_). We then input the generated sensor data (100 × 14 cases), which was unknown to this additional model. In light of our definition of ideal interpolated data (Figure 1B), we calculated within-individual correlations between the actual ($c_{\text{BF}\left(1\right)}^{\prime }$) and estimated (${ĉ}_{\text{BF}\left(1\right)}^{\prime }$) degrees of virtual openness for each participant. If the modulation of generated sensor data features by virtual openness ($c_{\text{BF}\left(1\right)}^{\prime }$) and the modulation of real sensor data features by real openness (*c*_BF(1)_) share similarities, we should see a strong positive correlation between the actual (*ĉ*_BF(1)_) and estimated (${ĉ}_{\text{BF}\left(1\right)}^{\prime }$) degrees of virtual openness in the real and generated data test.

**Figure 9:**
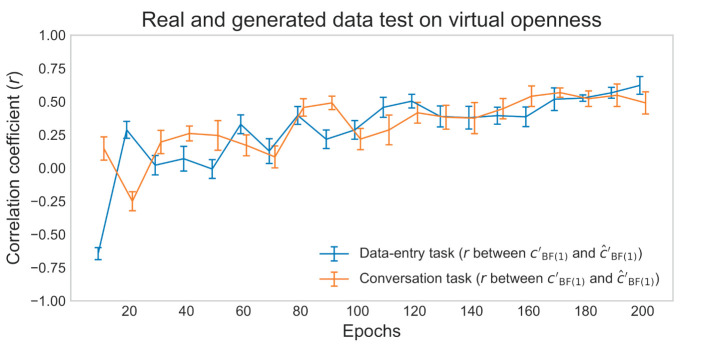
Mean correlation coefficients for data-entry and conversation tasks in the real and generated data test. Error bars represent SD.

**Figure 10:**
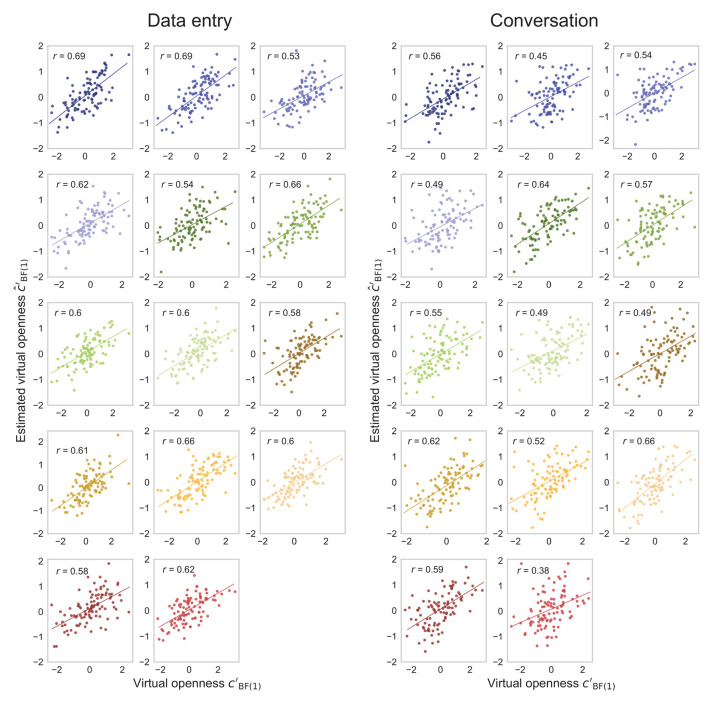
Fourteen scatterplots for data-entry and conversation tasks in the real and generated data test. Points represent openness values for each piece of sensor data, and different colors represent points of different individuals.

The actual ($c_{\text{BF}\left(1\right)}^{\prime }$) and estimated (${ĉ}_{\text{BF}\left(1\right)}^{\prime }$) degrees of virtual openness showed a strong within-individual positive correlation of about *r* = 0.5*-*0.6 in the later epochs of the real and generated data test (Figure 9). Scatterplots of the actual ($c_{\text{BF}\left(1\right)}^{\prime }$) and estimated (${ĉ}_{\text{BF}\left(1\right)}^{\prime }$) degrees of virtual openness in the final epoch are shown in Figure 10 (data entry: mean *r* = 0.61 [*SD* = 0.05]; conversation: mean *r* = 0.54 [*SD* = 0.07]). Again, no statistical tests were performed (see Section 2, Paragraph 4).

### 2.3 Discussion

To address the research gap, we conducted personality modulation analysis using a few participants in realistic situations. As the first contribution of the current study, we proposed the method of generating interpolated gaze behavior for this purpose. Overall, the results of the three evaluation tests suggest that our ss-InfoGAN successfully generated the sensor data for all participants while arbitrarily changing the degree of openness but keeping other individual attributes unchanged, or the interpolated sensor data (cf. Figure 1B).

The real data test examined whether the real Big Five traits modulated real gaze behavior. We confirmed that the discriminator path trained to estimate real openness from real sensor data of known participants could estimate openness of unknown participants in data-entry and conversation tasks. These results suggest that openness modulates gaze behavior in realistic data-entry and conversation tasks. Furthermore, we observed no correlation in the baseline task, with the same implementation and measurement. This suggests that the correlations in the data-entry and conversation tasks were not derived from errors in measurement or implementation.

The generated data test examined whether virtual openness modulated generated gaze behavior. We confirmed that the discriminator path involving the estimation of real openness could estimate virtual openness from generated sensor data within each participant in data-entry and conversation tasks. These results suggest that virtual openness modulates gaze behavior in generated sensor data even while keeping individual attributes fixed.

The real and generated data test examined whether the above two modulations share similarities. We confirmed that an additional discriminator, trained only to estimate real openness from real sensor data between individuals, could estimate virtual openness from the generated sensor data within each participant. These results suggest that openness modulations have similar effects on gaze behavior with real and generated sensor data.

Most importantly, these results collectively suggest that we successfully generated ideal interpolated data. We defined the ideal dataset as that interpolated from the minimum to the maximum degree of the target personality trait of openness for all individuals (Figure 1B). Accordingly, we conducted three tests in which the real data training was conducted on a between-individual basis, while the generated data estimation was conducted on a virtually within-individual basis. The discriminator-trained relationships were between real openness per individual and real sensor data per individual. In contrast, the discriminator-estimated relationships were between the changes in virtual openness within an individual and the changes in generated sensor data virtually within an individual. Similarities of openness modulations between real and generated sensor data in these tests suggest that the target personality trait of openness might be arbitrarily changeable during fixing of individual attributes in our ss-InfoGAN. Therefore, we conclude that our ss-InfoGAN successfully generated interpolated gaze behavior.

**Figure 11:**
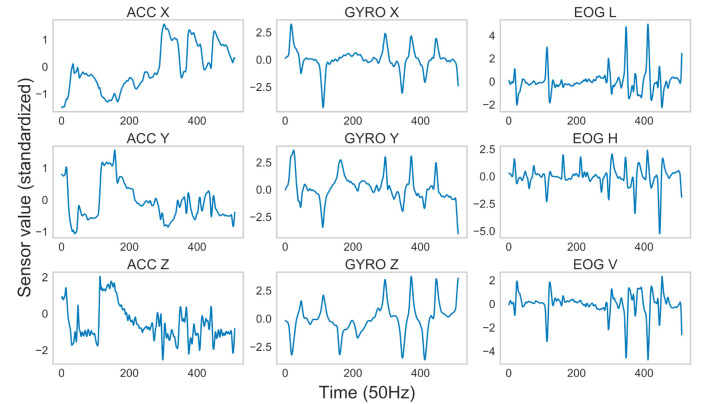
Example of real sensor data.

**Figure 12:**
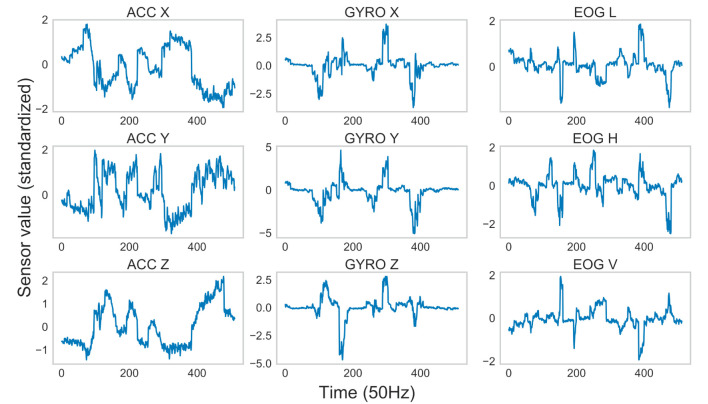
Example of generated sensor data.

Typical examples of real and generated sensor data are shown in Figures 11, 12. The figures indicate that the generated data preserved important properties of the time series of the real sensor data from head and eye movements. As accelerometers are sensitive to gravity, ACC values seemed to monitor the head orientation relative to gravity (head posture), as in previous studies (Meyer et al., 2018). For the GYRO values, transient increases/decreases were detected when rotations of the head posture occurred, sometimes in association with eye movements (Sağlam et al., 2011). As for the EOG values, only transient increases/decreases, reflecting saccades (i.e., rapid eye movements), were detected, as in previous studies (Bulling et al., 2010). The relationships across modalities were also preserved. For example, the patterns for ACC X had a shape that was roughly inverted in terms of the magnitudes of sensor values from ACC Y, which were similar to those from ACC Z. These relationships are thought to depend on the spatial arrangement of the sensors. We confirmed that a lack of variations in the generated sensor data caused by the failure of GAN training (i.e., “mode collapse”) (Bau et al., 2019) did not occur.

## 3 Experiment 2

As the second contribution of the current study, Experiment 2 evaluates the effectiveness of interpolated gaze behavior in real-world personality modulation analysis of a small number of participants in realistic situations. In line with the interactionist theory, we expected to find situation-dependent personality modulations of gaze behavior. Recent interactionists have suggested that personality traits modulate behaviors differently but systematically according to real-world situations (Fleeson, 2004, 2007; Funder, 2006; Baumert et al., 2017; Schmitt and Blum, 2020). Furthermore, personality traits may also modulate gaze behavior differently in realistic situations (Kaspar and König, 2012). However, it was not previously possible to analyze personality modulation of gaze behavior in realistic situations (Kröger et al., 2020).

In this evaluation, we expected that feature differences in the sensor data would be more clearly observed in the interpolated gaze behavior than in the real gaze behavior. We suspected that personality modulation analysis of real sensor data would be affected by individual attributes, as the differences due to the degree of a target personality trait (e.g., openness) may include those due to individual attributes (Figure 1A). In contrast, interpolated (generated) sensor data may enable the comparison of differences due to the degree of a target personality trait (e.g., openness) after excluding the possibility of bias in individual attributes (Figure 1B). Therefore, we expected a clearer feature change according to the degree of openness in the generated (interpolated) sensor data than in the real (sparse and biased) sensor data.

From the various features of head and eye movements (Liversedge and Findlay, 2000; Bulling et al., 2010; Fang et al., 2015; Steil and Bulling, 2015), we selected a very basic feature that could be tested for interpretation consistent with previous studies. Specifically, we calculated peaks of the autocorrelation coefficient of these movements, which is the correlation between the value at one point in time-series data and the value at a different point. The peaks reflect the period of the time-series data and might then include, for example, the saccade frequency (mean duration of fixations) in sensor data. As noted in the Introduction, one previous study has suggested that individuals with higher curiosity, a component of openness, tend to make frequent saccades in scene viewing (Risko et al., 2012), while another has reported that those with higher openness tend to make infrequent saccades to abstract animations (Rauthmann et al., 2012). We did not have strong hypotheses on how openness modulations of saccade frequency would differ between data-entry and conversation tasks because of the lack of analyses of gaze behavior in realistic tasks. Nevertheless, we at least expected that interpretations tied to these previous reports would be possible.

### 3.1 Materials and methods

#### 3.1.1 Data collection

We used the data from data-entry and conversation tasks collected in Experiment 1.

#### 3.1.2 Data generation

We used the ss-InfoGAN trained in Experiment 1. We generated sensor data for all 14 participants (virtual person-ID; $c_{\text{P}\left(1\text{-}14\right)}^{\prime }$) over the randomly sampled elapsed time (virtual time; $c_{\text{T}}^{\prime }$) while arbitrarily changing the degree of openness (virtual openness; $c_{\text{BF}\left(1\right)}^{\prime }$). We repeatedly generated 100 sets of sensor data for each degree of virtual openness ($c_{\text{BF}\left(1\right)}^{\prime }$), which was changed from an approximately minimum value of -2 to a maximum value of 2 in increments of 0.4. The latent codes corresponding to the other Big Five traits ($c_{\text{BF}\left(2\text{-}5\right)}^{\prime }$) were fixed at 0. The latent codes corresponding to the person-ID and time ($c_{\text{P}\left(1\text{-}14\right)}^{\prime }$ and $c_{\text{T}}^{\prime }$) were fixed but sampled in the same way as during training to eliminate differences in individual attributes.

### 3.2 Results

The average autocorrelation coefficients were calculated for each degree of openness from the time series of the overall amplitudes of head and eye movements. We first smoothed the generated sensor data, as we did with the real sensor data in Experiment 1. Only the accelerometer indicates the absolute direction of the head, while the gyrometer and EOG produce time-series data reflecting the relative vertical and horizontal motion of the head or eye. The accelerometer data was then differentiated and transformed to the relative motion of the head direction. Since all of these relative motions were standardized to an average value of 0, their absolute values could be taken to obtain time-series data representing the degree to which motion occurred, regardless of the direction of motion (up, down, left, or right). Accordingly, the overall amplitudes were obtained by adding the above absolute value of the time series of six sensor data components (ACC X, Y; GYRO X, Y; EOG H, V) on a vertical plane relative to the gazing object in front of the head/eye.

**Figure 13:**
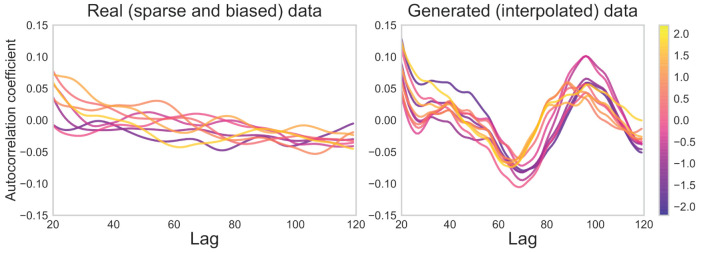
Autocorrelation coefficients for real (sparse and biased) and generated (interpolated) sensor data for data-entry task. Low-openness data are shown in purple and high-openness data in yellow. The value of one lag unit corresponds to one unit of time in the 50 Hz sensor data.

For the data-entry task, as shown in Figure 13, the autocorrelation coefficients for each openness $c_{\text{BF}\left(1\right)}^{\prime }$ were highly disparate in the real (sparse and biased) data, with only a slight downward trend in the autocorrelation coefficient as lag increased. In contrast, the coefficient value peaked in the lag range of 80*-*100 in the generated (interpolated) data. Also, the lag with these peaks decreased with increasing openness.

**Figure 14:**
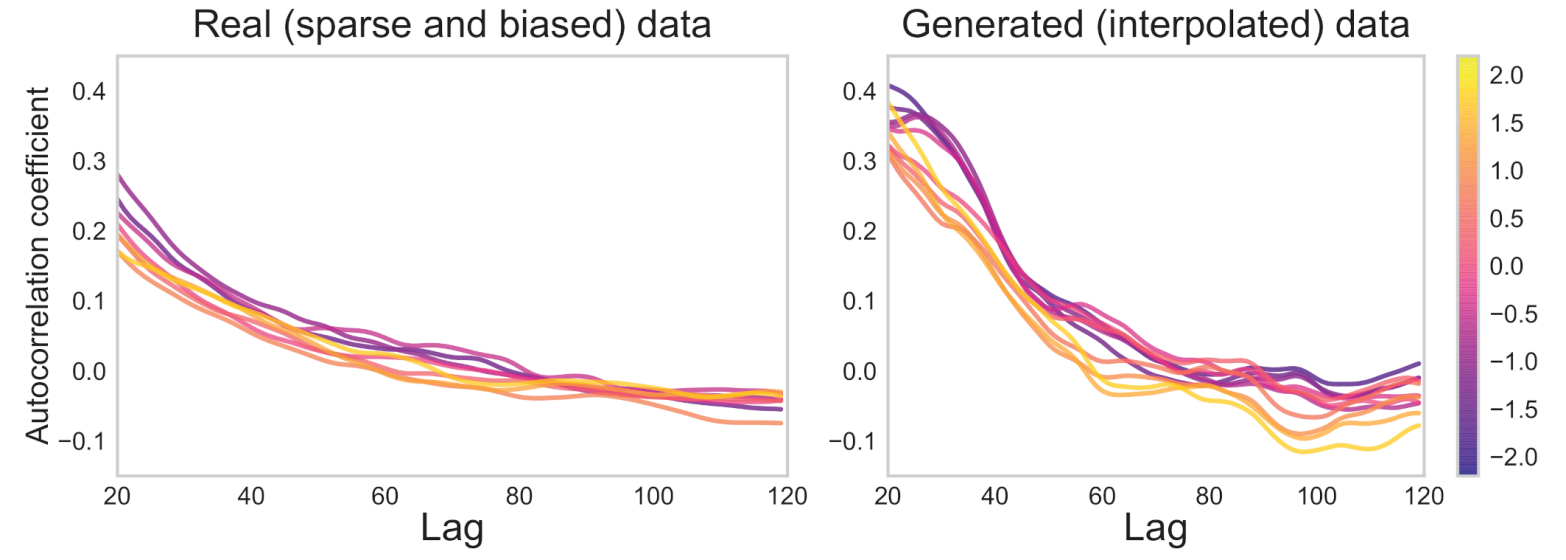
Autocorrelation coefficients for real (sparse and biased) and generated (interpolated) sensor data for conversation task. Low-openness data are shown in purple and high-openness data in yellow. The value of one lag unit corresponds to one unit of time in the 50 Hz sensor data.

For the conversation task, as shown in Figure 14, values that varied slightly with openness were barely visible between lags 20*-*80 in the real data. In contrast, as openness increased, the peak of the coefficient in the lag range of 20*-*40 shifted to around 80 (or vanished) in the generated data. Note that no statistical tests were performed due to the arbitrarily large sample size of generated sensor data (see Section 2, Paragraph 4).

### 3.3 Discussion

As the second contribution of the current study, Experiment 2 evaluated interpolated gaze behavior in terms of real-world personality modulation analysis. Previous studies on this topic controlled experimental situations and used large numbers of participants (Isaacowitz, 2005; Rauthmann et al., 2012; Risko et al., 2012; Lea et al., 2018; Kröger et al., 2020; Rubo et al., 2023), suggesting the difficulty in detecting real-world personality modulations (Figure 1A) (Kröger et al., 2020). Indeed, in the current study, we failed to find feature differences (autocorrelation differences) associated with openness in the real (sparse and biased) sensor data of 10*-*20 people in realistic data-entry versus conversation tasks. Recognizing these difficulties, we alternatively analyzed interpolated gaze behavior (Figure 1B) (Vinciarelli and Mohammadi, 2014; Junior et al., 2019; Phan and Rauthmann, 2021). Consequently, we did find task differences associated with openness in the generated (interpolated) sensor data. It is crucial that Experiment 1 already showed that these feature differences in the sensor data generated by changing the degree of virtual openness reflect differences in the real sensor data due to the degree of real openness. That is, we found the interpolated sensor data to be effective in personality modulation analysis even with a small number of participants in realistic situations.

These results suggest that openness may modulate real-world gaze behavior situation-dependently. The lag with the peak coefficient was reduced with increasing openness for the data-entry task. The coefficients suggest that immediately after a head or eye movement (e.g., saccade) occurs once, subsequent movements are unlikely to occur again (e.g., fixation) (cf. Just and Carpenter, 1976; Bulling et al., 2010; Sağlam et al., 2011), resulting in a negative coefficient in the lag range of 60*-*80. After this period, the possibility of eye movements occurring again might arise, with the average period of these eye movements appearing as the peak of the autocorrelation coefficient in the lag range of 80*-*100. Accordingly, the current results suggest that the saccade frequency (mean fixation duration) increased (decreased) with increasing openness in the data-entry task. These findings may be consistent with a previous study using a scene-viewing task (Risko et al., 2012).

Such a clear repetitive saccade-fixation pattern did not occur for the conversation task. Nevertheless, the coefficient peak at the shorter lag shifted to a longer lag or vanished as openness increased. Accordingly, the saccade frequency (mean fixation duration) may have decreased (increased) with increasing openness in the conversation task. They may be consistent with a study using an abstract animation (Rauthmann et al., 2012).

Given these, we theoretically propose that openness may modulate gaze behavior depending on the type of cognitive processing required to perform the task. Previous studies have shown that eye movements change depending on the external or internal attention (Smilek et al., 2010; Annerer-Walcher et al., 2021). Also, significant relationships have been suggested between openness and these types of attention (Marty-Dugas and Smilek, 2019). Accordingly, when the main task is processing external visual stimuli, such as scene viewing in a previous study (Risko et al., 2012) and data entry in the current study, higher openness may lead to a more liberal switching of the gazing location with frequent spatial and temporal frequency. In contrast, when the main task is thinking internally, such as watching an abstract animation (i.e., interpreting meaningless visual stimulus) in a previous study (Rauthmann et al., 2012) and conversation (i.e., gathering auditory information and making responses) in the current study, higher openness may increase the fixation duration at each fixation point. The longer duration may reflect the deepened cognitive processing of the gazing object (Just and Carpenter, 1976; Siegenthaler et al., 2014). Taken together, the consideration that openness modulates the shortening or lengthening of fixation time, depending on external and internal processing, is a hypothesis worth considering. Future studies are warranted to examine this hypothesis in a more comprehensive range of situations. Extensive future studies have the potential to reveal the complete picture of situation-specific personality modulations of gaze behavior in real-world activities.

## 4 General discussion

Despite the importance of real-world personality modulation of gaze behavior, previous methods of investigation have required unrealistic situation control and large numbers of participants (cf. the research gap). We assumed that these shortcomings may have been attributable to the sparse and biased nature of gaze behavior, and we addressed the research gap by investigating interpolated gaze behavior. The first contribution of this study was to provide a methodology for generating interpolated sensor data (Experiment 1). The second contribution was to evaluate the effectiveness of the interpolated (generated) data in analyses of a small number of participants in realistic situations (Experiment 2).

Experiment 1 suggests that interpolated gaze behavior could be the first step in real-world personality modulation analysis. As previously noted, previous methods in this field have required controlled situations and a large number (50*-*250) of participants, which prevented real-world analysis (Isaacowitz, 2005; Rauthmann et al., 2012; Risko et al., 2012; Lea et al., 2018; Rubo et al., 2023). Other studies in related fields that have used estimation rather than analytical techniques have also produced comparable effects in similar settings (Hoppe et al., 2015, 2018; Cuculo et al., 2018; Berkovsky et al., 2019; Millecamp et al., 2021; Sarsam et al., 2023). Several studies estimated with high accuracy the degrees of the Big Five traits in well-controlled situations (Cuculo et al., 2018; Berkovsky et al., 2019; Sarsam et al., 2023). The remaining studies estimated them from gaze behavior in realistic tasks only with low accuracy (i.e., accuracy significantly higher than chance level) (Hoppe et al., 2015, 2018; Millecamp et al., 2021). In light of these previous studies, we conclude that the significant openness modulation represented in sensor data generated from our 14 participants in realistic situations provided an effective methodology for personality modulation analysis, which has not been previously established.

The interpolated gaze behavior helps elucidate how gaze behavior explicitly differs depending on personality in real working situations, in contrast to studies that only implicitly estimated personality traits from gaze behavior (Hoppe et al., 2015, 2018; Cuculo et al., 2018; Berkovsky et al., 2019; Millecamp et al., 2021). Three estimation studies used a “black-box” model, which implicitly detected a relationship between the input (gaze behavior) and the output (personality traits) (Hoppe et al., 2015; Berkovsky et al., 2019; Millecamp et al., 2021). Although this model demonstrated that personality traits can be estimated from gaze behavior, it did not explicitly indicate what kind of relationship exists. Additional studies showed which features in the input (gaze behavior) contribute to estimating the output (personality traits), but they did not define relationships between the two for further analysis (Cuculo et al., 2018; Hoppe et al., 2018). In contrast to these studies, our approach not only implicitly detected the relationship between the input (gaze behavior) and the output (openness) but also represented it in the interpolated sensor data. Since the generated sensor data mimicked the raw, real sensor data, we were able to explicitly analyze the represented personality modulation using the generated sensor data.

Experiment 2 suggests that the interpolated gaze behavior can reveal how openness systematically modulates cognitive inputs depending on realistic working situations. Recent interactionist studies have shown that the Big Five traits do not change over the long term, but the extent to which personality traits modulate behavior varies across situations (Fleeson, 2004, 2007; Funder, 2006; Baumert et al., 2017; Schmitt and Blum, 2020). In previous studies, researchers analyzed situation-dependent personality modulation behavior using “experience sampling,” in which participants' behavior is collected through subjective responses to questions asked every several hours. Thus, the questions are relatively simple, and it is not easy to examine what kind of cognitive processing is responsible for behavior modulation. In contrast, personality modulation analysis of gaze behavior in realistic situations reveals how personality traits modulate gaze behavior associated with overt visual spatial attention in cognitive processing (Kaspar and König, 2012). The current results suggest that openness situation-dependently modulates cognitive inputs, which has not been previously demonstrated.

The concept of interpolated gaze behavior may ultimately resolve the difficulty in analyzing systematic, situation-dependent personality modulation of real-world cognitive processing. Personality traits, especially the Big Five, are psychological constructs that have been identified to explain individual differences based on subjective verbal reports of behavioral tendencies in everyday life (Allport and Odbert, 1936; McCrae and Costa, 1987, 2008; Goldberg, 1990). In contrast, overt visual spatial attention (i.e., gaze behavior) has often been investigated in well-controlled cognitive psychological experiments in which individual differences are to some degree eliminated (Hedge et al., 2018). Personality modulation analysis of gaze behavior in realistic, everyday situations may help to bridge this gap. Interpolative generation of other areas of behavior data, including gaze analysis in a broader range of situations, should be helpful in advancing the theoretical understanding of the interactionist view of personality traits and practical applications, such as the realization of personalized assignments of work-related tasks (real-life situations).

### 4.1 Limitations and future directions

The primary limitation of the current method is its inability to capture the relationships between the target personality trait and the sensor data features beyond the structure of the real dataset's latent codes (personality traits and individual attributes). Our ss-InfoGAN works only when a target personality trait is continuously varied, and individual attributes are varied discontinuously and orthogonally to that trait. If the individual attributes are continuously aligned to the degree of the target personality trait in the real sensor data, our ss-InfoGAN inevitably reproduces such biases on the generated sensor data. For example, suppose that the degree of openness for each participant is strongly positively correlated with age. In that case, it is inevitable that differences in gaze behavior caused by aging would be reproduced in the generated sensor data as reflecting openness modulation. This should be kept in mind when conducting personality modulation analysis using behavior data generated by interpolation.

Several procedural limitations can be pointed out. Among these is the accuracy of sensor devices. We used a wearable sensor device called JINS MEME, which is similar in size to ordinary glasses, to ensure that the participants' subjective sensation of the experimental situation was not significantly different from real-life situations. JINS MEME has been reported to measure gaze behavior with sufficient precision for our current purpose, capturing very basic eye movement events such as saccades during daily activities (Ishimaru et al., 2014, 2016, 2017). Nevertheless, accurate detection of fine features of eye movements, such as those that appear only at temporal resolutions below 20 ms (1000 ms/50 Hz) (e.g., microsaccades) may be difficult with such an apparatus (Laubrock et al., 2005; Jazbec et al., 2006). Future studies are warranted to examine the effectiveness of our ss-InfoGAN with finer eye movement data.

We can not completely rule out the possibility that temporal variations during experiment execution influenced the current results. For example, comfort with or fatigue due to the experiment, such as comfort with the use of wearable sensors, may increase over time, so there may be differences between tasks performed in the first and in the second half of the session. We performed most tasks in random order, and only the conversation task was fixed at the end. Feature differences in the head and eye movements between the data-entry and conversation tasks could be due, in part, to the fact that comfort or fatigue was greater in the conversation task than in the data-entry task. Further, openness modulation in the conversation task may incorporate differences in this comfort/fatigue maximum according to degree of openness. Since our ss-InfoGAN can, in theory, interpolate sensor data according to the elapsed time from the start of the experiment, future studies using our technique to mitigate other variations such as temporal variation are expected.

Finally, we examined only basic features (autocorrelation coefficients) of gaze behavior to determine the effectiveness of the interpolated sensor data; more advanced features were not extracted. Analysis of various features of gaze behavior in real-life situations should be a fruitful future direction. For example, previous studies have shown that supervised (Bulling et al., 2010) or unsupervised (Steil and Bulling, 2015) machine learning methods can detect macro behaviors such as computer work and media viewing, as well as moderate behaviors such as reading text (Kunze et al., 2013). Moreover, rule-based methods can detect micro behaviors such as head-eye coordination (Fang et al., 2015), fixations (Just and Carpenter, 1976), and saccades (Liversedge and Findlay, 2000), which are associated with specific cognitive processes. Understanding personality modulation of gaze behavior from a broader perspective should provide further insights into the theoretical understanding and practical applications of personality-modulated overt visual spatial attention (Alves et al., 2020).

### 4.2 Conclusion

The current study aimed to fill the research gap where we could not empirically show the theoretically expected personality modulation of real-world gaze behavior. As the first contribution, we provided a methodology for generating gaze data with the separation of individual attributes and personality traits, which addressed the cause of the research gap. As the second contribution, this generated sensor data revealed the expected situation-dependent openness modulation of real-world gaze behavior even from head and eye movements obtained from only 14 participants performing realistic tasks. We conclude that our method has the potential to expand our insights into real-world, systematic personality modulation of gaze behavior depending on the situation.

## Author's note

Note that the current study used data from our previous report that addressed the engineering research question in the computer science field (Yamashita et al., 2022). While the previous report did not primarily address the psychological research question, the current study has tested the hypothesis of interactionist psychology for the first time. That is, the current study has examined personality modulation of real-world gaze behavior varies in a situation-dependent manner.

## Data availability statement

The original contributions presented in the study are included in the article/supplementary material, further inquiries can be directed to the corresponding author.

## Ethics statement

The studies involving humans were approved by the Ethics Committee, Graduate School of Informatics, Kyoto University (KUIS-EAR-2019-004). The studies were conducted in accordance with the local legislation and institutional requirements. The participants provided their written informed consent to participate in this study.

## Author contributions

JY and TK designed the study. HO and TK oversaw the study. TK collected the data. JY implemented and evaluated the proposed method, generated all results and figures, and wrote the paper. YT advised on the analyses. All authors revised the manuscript, approved the manuscript for publication, and agreed to be accountable for all aspects of the work to ensure that questions related to the accuracy or integrity of any part of the work are appropriately investigated and resolved.

## Footnotes

^1^Because of time constraints, we collected valid data for only 13 participants in the baseline task.

^2^https://github.com/Natsu6767/InfoGAN-PyTorch, https://github.com/fjxmlzn/InfoGAN-CR (accessed May 22, 2023).
